# IS BODY MASS INDEX AND OBESITY SURGERY MORTALITY SCORE IMPORTANT IN PERIOPERATIVE COMPLICATIONS OF LAPAROSCOPIC SLEEVE GASTRECTOMY BEFORE DISCHARGE?

**DOI:** 10.1590/0102-672020210002e1602

**Published:** 2021-10-18

**Authors:** Mehmet Buğra BOZAN, Nizamettin KUTLUER, Ali AKSU, Ayşe AZAK BOZAN, Burhan Hakan KANAT, Abdullah BÖYÜK

**Affiliations:** 1 Department of Surgery, Kahramanmaras Sutcu Imam University, Professor (Assistant), Kahramanmaras, Turkey; 2 Department of Surgery, Elazig Training and Research Hospital, Specialist of General Surgery, Elazig, Turkey; 3 Department of Anesthesiology and Reanimation, Elazig Training and Research Hospital, Specialist of General Surgery, Elazig, Turkey; 3 Department of Surgery, Malatya Turgut Özal University, Professor (Associate), Malatya, Turkey; 4 Department of Surgery, Elazig Training and Research Hospital, Professor, Elazig, Turkey

**Keywords:** Morbid obesity, Laparoscopic sleeve gastrectomy, Perioperative complication, Obesity surgery mortality risc score, Obesidade mórbida, Gastrectomia vertical laparoscópica, Complicação perioperatória, Escore de risco de mortalidade em cirurgia de obesidade

## Abstract

**Background:**

Morbid obesity surgery and related complications have increased with time.

**Aim:**

To evaluate the relationship between perioperative complications before discharge and preoperative body mass index and obesity surgery mortality score in laparoscopic sleeve gastrectomy.

**Method:**

1617 patients who met the inclusion criteria were evaluated retrospectively. The patients were examined in terms of demographic data, presence of comorbidities, whether there were complications or not, type of complications and obesity surgery mortality score.

**Results:**

Complications were seen in 40 patients (2.5%) and mortality wasn’t seen in the early postoperative period before discharge. The mean age of patients with complications was 36.3±10.02 years (19-57) and without complications 34.12±9.54 (15-64) years. The preoperative mean BMI values of patients with and without complications were 45.05±3.93 (40-57) kg/m^2^ and 44.8±3.49 (35-67) kg/m^2^ respectively. According to BMI groups 40-45 kg/m^2^, 45-50 kg/m^2^ and 50 and over, there was not any statistical significance seen in three groups in terms of complication positivity and major-minor complication rates. There was not any statistical significance seen between the patients with and without major-minor complications and obesity surgery mortality score.

**Conclusion:**

There was not any relation between perioperative laparoscopic sleeve gastrectomy complication rates before discharge and BMI and obesity surgery mortality scores.

## RESUMO

***Racional*:**

A cirurgia da obesidade mórbida e complicações relacionadas aumentaram com o tempo.

**Objetivo:**

Avaliar a relação entre as complicações perioperatórias antes da alta e o índice de massa corporal pré-operatório e o escore de mortalidade da cirurgia de obesidade na gastrectomia vertical laparoscópica.

**Método:**

1.617 pacientes que atenderam aos critérios de inclusão foram avaliados retrospectivamente. Os pacientes foram examinados quanto aos dados demográficos, presença de comorbidades, ocorrência ou não de complicações, tipo de complicações e escore de mortalidade cirúrgico da obesidade.

**Resultados:**

Complicações foram observadas em 40 pacientes (2,5%) e mortalidade não foi observada no período pós-operatório imediato antes da alta. A média de idade dos pacientes com complicações foi de 36,3±10,02 anos (19-57) e sem complicações de 34,12±9,54 (15-64) anos. Os valores médios de IMC pré-operatórios dos pacientes com e sem complicações foram 45,05±3,93 (40-57) kg/m^2^ e 44,8±3,49 (35-67) kg/m^2^, respectivamente. De acordo com os grupos de IMC 40-45 kg/m^2^, 45-50 kg/m^2^ e 50 e mais, não houve qualquer significância estatística observada em três grupos em termos de positividade de complicações e taxas de complicações maiores-menores. Não houve significância estatística entre os pacientes com e sem complicações maiores e menores e o escore de mortalidade da cirurgia de obesidade.

**Conclusão:**

Não houve qualquer relação entre as taxas de complicações da gastrectomia vertical laparoscópica perioperatória antes da alta e os escores de mortalidade da cirurgia de obesidade e IMC.

## INTRODUCTION

The term obesity is defined as body mass index (BMI) 30 and over, and morbid obesity is considered as BMI greater than 40^13^. Its incidence in the general population is approximately 20% according to Organisation for Data of Economic Co-operation and Development (OECD) countries and according to the recent data of the countries, its incidence has reached 20%. Unfortunately, it is increasing worldwide^22^. Obesity should not be thought of as a single disorder as it is related to many disorders like hypertension, diabetes, obstructive sleep apnea, cardiovascular diseases, metabolic alterations, gastroesophageal reflux and increased risk of malignancies^9^ ^,^ ^13^ ^,^ ^28^ ^,^ ^29^ **.** For years people have struggled with obesity with both metabolic and physical problems. Surgical treatment is the most effective long-term therapeutic treatment in current and modern medicine of obesity and obesity-related diseases as the last resort^16^. Roux-en-Y gastrojejunostomy is the method that has been applied for many years and there is consensus on its effect. However, in recent years, laparoscopic sleeve gastrectomy (LSG) has an increasing number of procedures with a short learning curve and it is the most performed surgical technique all over the world and also in Turkey^22^ ^,^ ^30^. 

Unfortunately, like any surgical procedure, this surgery has its own complications. Although being performed frequently increases the experience of surgeons, this situation cannot reduce the risk of complications of surgery to zero. In morbid obesity patients, the risk of any complications in all surgical procedures is higher than with other patients who were not morbidly obese. Due to these complications, prolonged hospital stays, increased reapplications to the hospital, reoperations and deaths can result^4^ ^,^ ^19^. Despite both an increased risk of complications according to obesity and the risk of specific complications due to sleeve gastrectomy, laparoscopic sleeve gastrectomy is associated with acceptable postoperative morbidity and mortality rates^18^. 

Various classifications have been described in the literature for complications after surgery. In one of these classifications, according to Clavien-Dindo classification, complications are divided into two groups as major and minor^12^ ^,^ ^13^. This classification can be applied to bariatric and metabolic surgeries as with all surgery types. Especially major complications in this classification are life-threatening situations and their early detection is important^13^ ^,^ ^18^.

 In fact, surgeons do not want to encounter mortality in any of their patients. In this respect, DeMaria et al. developed an easily applicable mortality risk scoring system, which is consisted of five items (age ≥45 years, male gender, BMI ≥50 kg/m^2^, arterial hypertension, and risk factors for pulmonary thromboembolism, and can be used for the pre-operative determination of risky patients in obesity surgery, i.e., Obesity Surgery Mortality Risk Score (OR-MRS)^11^ ^,^ ^15^ ^,^ ^18^.

In this study, we aimed to determine the perioperative complications seen in the LSG patients that we performed in our clinic without being discharged from the hospital and to evaluate the treatment processes of the complications under literature. In addition, whether the OS-MRS risk assessment scale had a role in determining perioperative complications before discharge was investigated.

## METHOD

Our study was carried out with the approval number 13281952-929 from Elazig Training and Research Hospital. All procedures performed in studies involving human participants were in accordance with the ethical standards of the institutional and/or the national research committee and with the 1964 Helsinki declaration and its later amendments or comparable ethical standards. 

A total of 1,752 patients who met the criteria of patient selection in terms of obesity and metabolic disease surgery, were enrolled. The inclusion criteria were: 1) patients with BMI of 40 and above, without the additional comorbid disease; 2) patients with a BMI of 35 and above, with the additional comorbid disease, such as hypertension, diabetes mellitus; and 3) who were operated on in the Elazığ Training and Research Hospital General Surgery Clinic between January 2016 and October 2018. They were evaluated retrospectively. Patients’ data were obtained from epicrisis forms in the hospital computer system, patient follow-up charts, and patient files. Data for OS-MRS and Clavien Dindo complication classification were obtained from patient follow-up charts, patient files, and hospital computer records. Patients’ demographic data (age, gender), presence of comorbidities, complications (wound complications, thromboembolic events, staple line leakage, splenic infarction proven by imaging modalities, bleeding detected due to low hemoglobin and hematocrit values during follow-up, acute renal failure due to deterioration in biochemical parameters), complication type (major and minor), whether emergency surgery was performed, BMI values, postoperative hospitalization, and OS-MRS was recorded. Additionally, while grouping according to BMI values, patients with BMI below 40 were excluded and three groups with BMI values of 40-45 kg/m^2^, 45-50 kg/m^2^, and 50 kg/m^2^ and above were created. Whether there were any complications among these groups and the presence of major or minor complications by Clavien-Dindo classification was investigated. 

Exclusion criteria were: 1) patients’ data not available or the ones who were operated with other types of bariatric metabolic surgery; 2) patients who left the hospital due to referral; 3) patients whose OS-MRS scale didn’t be calculated; and 4) patients whose American Society of Anesthesiologists (ASA) score was 4 and greater. 

After using the exclusion criteria, 1,617 patients were enrolled.

### Statistical analysis

The power analysis of the study was conducted with the G-Power 3.0.10 programming system. The estimated power analysis for the sample size of this retrospective study had been shown that 1617 cases had 0.5 effect size, α: 0.05 and a power 0.88. IBM Statistical Package for Social Sciences (SPSS) 20.0 was used for statistical evaluation. Kolmogorov-Smirnov test results were examined in terms of the suitability of the groups for normal distribution. In comparisons between groups, an independent sample t-test or Mann Whitney U test was used to evaluate numerical data according to the normality test. In the evaluation of categorical data, chi-square analysis and Fisher’s exact test were performed. In terms of the relation between complication formation and BMI, univariate analysis and multivariate analysis were performed. Numerical data were given as mean±standard deviation (SD, minimum-maximum values) or median (minimum-maximum values) according to the normality test. Categorical data are given as count (n) and percentage (%).

## RESULTS

The male/female ratio was 317/1300 (19.6%/80.4%). The mean age of all patients was 34.18±9.56 (15-64) years and mean BMI was 44.81±3.5 (35-67) kg/m^2^. The mean hospitalization time was 4.22±1.69 (3-35) days for all patients. Demographic data and comorbidities of the patients are given in Table 1.

**TABLE 1:** Analysis of the demographic and clinical characteristics of the overall patients

Variable		
Gender (Female/Male)	317/1300 (19.6%/80.4%)	
Age (years)	34.18 ± 9.56 (15 - 64)	
BMI (kg/m^2^)	44.81 ± 3.5 (35 - 67)	
Preoperative one or more comorbidity (+/-)	546/1071 (33.8%/66.2%)	
	Arterial hypertension	104/1513 (6.4%/93.6%)
	Diabetes mellitus	297/1320 (18.4%/81.6%)
	OSAS	7/1610 (0.4%/99.6%)
	Hypothyroidism	57/1560 (3.5%/96.5%)
	PCOS	29/1588 (1.8%/98.2%)
	Asthma	52/1565 (3.2%/96.8%)
	Still disease	1/1616 (0.1%/99.9)
	Deep venous insufficiency	83/1534 (5.1%/94.9%)

BMI=body mass index; OSAS=obstructive sleep apnea syndrome; PCOS=polycystic over syndrome

The mean age of male was 34.46 ± 8.77 (16 - 58) years and the mean age of female patients was 34.11±9.74 (15-64) years. The mean BMI of males was 44.98±3.72 (37-60) kg/m^2^ and the mean BMI of females was 44.77±3.44 (35-67) kg/m^2^. There was not any statistical difference between males and females for age and BMI (p=0.525 and 0.368 respectively). 

**FIGURE 1:**
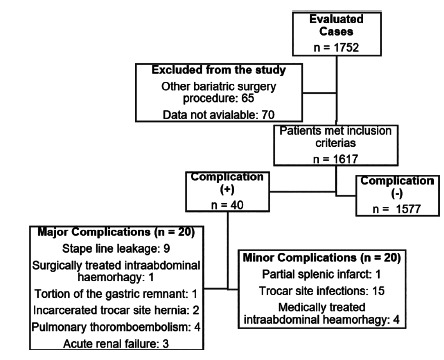
Flow chart of the complication distribution complication of laparascopic sleeve gastrectomy before discharge

**FIGURE 2:**
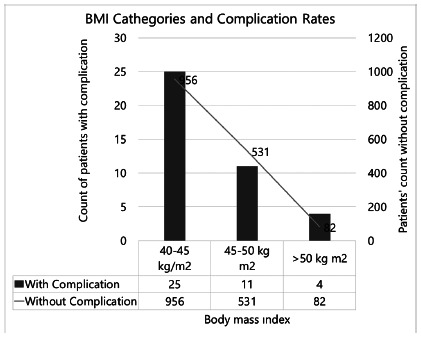
Complication rates according to the body mass index (BMI) groups

Perioperative complications were seen in 40 (2.5%) patients during hospitalization. Mortality was not seen in any of our patients in the early postoperative period before discharge. The mean age of these patients with complications was 36.3±10.02 (19-57) years and no statistical significance was seen when compared with the patients without complications (p=0.181). The mean BMI values of patients with complications and without complications were 45.05±3.93 (40-57) kg/m^2^ and 44.80±3.49 (35-67) kg/m^2^, respectively (p=0.686, r^2^: -0.001). There was not any statistically significance in complication rates and Clavien-Dindo major/minor complication rates between BMI dependent groups (BMI 40-45 kg/m^2^, 45-50 kg/m^2^ or 50 and greater, p=0.737 and 0.492 respectively, Figures 1 and 2). 

Twenty minor (wound infections, partial splenic infarct, intra-abdominal hemorrhage due to staple line controlled with transfusion) and 20 major (anastomosis leakage, intra-abdominal haemorrhage controlled with surgical intervention, early trocar side hernias, torsion of the gastric remnant, pulmonary thromboembolism, acute renal failure) were seen as complications (Table 2). OS-MRS scale rate of the patients with complications was 36 patients in Class A and four in Class B. There was not any statistical significance seen between the OS-MRS scale and complications seen in patients and between OS-MRS and the Clavien-Dindo Complication Scale (p=0.275 and 0.13 respectively, Table 3). 

**TABLE 2:** The distribution of perioperative complications before discharging according to Clavien Dindo Classification of surgical complications.

Clavien Dindo Classification	Count (n)	Percentage (%)	Complication Type	n
Minor complications				
I	16	0.99%		
			Partial splenic infract	1
			Trocar site infection	15
II	4	0.25%	Intraabdominal hemorrhage from staple line	4
Total	20	1.24%		
Major complications				
III	13	0.8%		
IIIA	7		Staple line leakage	7
IIIB	6		Staple line leakage	2
			Intraabdominal hemorrhage from staple line	1
			Torsion of the gastric remnant	1
			Trocar site hernia	2
IV	7	0.43%		
IVA	7		Pulmonary thromboembolism	4
			Acute renal failure due to rhabdomyolysis and acute tubular necrosis	3
IVB	0			
V	0			
Total	20	1.24%		

**TABLE 3:** Complication positivity and Clavien Dindo classification of surgical complications with obesity surgery mortality score (OS-MRS)

Variable	Complication (+)	No Complication	***p* value**	CD I-II (Minor)	CD≥ III (Major)	p
OS-MRS			0.275			0.13
Class A	36 (2.2%)	1399 (86.5%)		18 (1.2%)	18 (1.2%)	
Class B	4 (0.2%)	173 (10.7%)		2 (0.1%)	2 (0.1%)	
Class C	0 (0%)	5 (0.3%)		0 (0%)	0 (0%)	
Total	40 (2.5%)	1577 (87.5%)		20 (1.2%)	20 (1.2%)	

While the duration of hospitalization time of patients with complications was 11.3±6.48 (4-35) days, the duration of hospitalization time of patients without complications was 4.04±0.77 (3-10) days (p<0,001). 

## DISCUSSION

Although laparoscopic morbid obesity surgery has satisfactory metabolic results, serious lethal complications can be observed in patients after surgery. Complications specific to bariatric surgery types vary. These complications are divided into two groups: early/perioperative complications are seen in the postoperative first month, and late complications seen after the first month of surgery. The development time of postoperative complications cannot be predicted, however the vast majority of complications that can cause medical problems to appear before discharge^20^. For this reason, it is important to distinguish the perioperative complications that occur before discharge from the perioperative complications that occur after discharge.

LSG has its specific complications. In addition to staple line leaks, complications such as intra-abdominal bleeding of the staple line or trocar site, intra-luminal bleeding of the staple line, trocar site herniations, pulmonary complications (thromboembolism, atelectasis and, accordingly, pneumonia) are life-threatening complications seen in LSG^7^. Morbidity rates in the first 30 days postoperatively are 5%, while mortality is 0.11%^2^. Patients in our study were followed up in the first three days of this process. While no mortality was observed in any of our patients (0%), our morbidity rates were lower than in the literature (2.5%). We thought that our lower morbidity and mortality rates were only due to be perioperative rates before discharge.

Early staple line leaks, one of the major complications causing an increase in mortality and morbidity, are seen in the literature at the rate of 0-5%^13^. Early perioperative leaks are mostly caused by problems in the proper placement of the staple line and are associated with problems in the surgical technique. Clinically, early tachycardia is the most common symptom. In addition, frequent breathing and respiratory distress preclude leaks^20^. In addition to weakness and fatigue, if abscess formation occurs, fever, leucocytosis, and exacerbated signs of infection will appear. With the progression, abdominal distention, tenderness, and a rarely handled mass may occur. If the leakage occurs towards the lung, it will cause pulmonary complications (such as atelectasis, lower basal zone pneumonia)^20^. In terms of detection of leakage, tomography with water-soluble oral contrast material will guide the patients. When leakage is detected, oral intake is stopped, as is done in our patients, and since it is thought to be caused by a mechanical failure in the early period, surgical drainage methods and reconstructions can be performed, or an alternative interventional radiological drainage and observation can be performed in addition to nutritional support^20^. Although small leaks can be closed spontaneously, laparoscopic drainage methods can be used if the leak cannot be controlled^5^ ^,^ ^24^. In our study, staple line leakage was observed in nine patients (0.6%).

Hemorrhage from the staple line into the abdomen or gastrointestinal tract is the most common non-septic complication in the postoperative period of LSG^20^. Intra-abdominal haemorrhage was more frequently seen in inadequate hemostasis performed patients. Silecchia et al reported that bleeding rates ranged from 0-20% in the review articles in which they examined postoperative sleeve gastrectomy complications and this increased the reoperation rates^27^. However, it is important to decide which patients will be re-operated due to patient’s clinical findings (weakness, fatigue, tachycardia, hypotension), if the drain is actively working, the amount and character of the drainage material and how much transfusion will be needed. Adhering to massive blood transfusion rules can prevent early and unnecessary surgical interventions^8^. In our study, five of our patients (0.3%) had intra-abdominal bleeding due to stapler line bleeding. The median daily blood and blood product replacement requirement was four units blood and blood products (3-8 units). 

The risk of developing pulmonary thromboembolism has increased with morbid obesity, even if not from morbid obesity surgery. These rates range from 0% to 0.4% in bariatric surgery patients^31^. In the literature, early pulmonary thromboembolism rates were reported as 0.25% in a metanalysis reported by Chang et al.^4^ Contival et al similarly stated these rates being between 0% and 0.6%^7^. For treatment, patients should be taken to the intensive care unit, followed closely, low molecular weight heparin should be started and they should be treated with nasal oxygen. If thromboembolic events are severe, the need for intubation may occur^20^. In our study, pulmonary thromboembolism was observed in 0.2% (n=4).

Early trocar site hernias are rare major complications of sleeve gastrectomy encountered in the clinic. In their study evaluating major complications of sleeve gastrectomy, Debs et al reported that trocar site herniations were seen in one patient out of 434^10^. Similarly, only two of 1,617 had early trocar site herniation in our study (0.1%). 

Rarer perioperative complications include spleenic infarction and wound site infections as minor complications and acute tubular necrosis due to rhabdomyolysis and consequently acute renal failure as major complications. Debs et al followed splenic infarction in only two of 434 patients in their studies (0.46%)^10^. Splenic infarction is generally seen as a partial infarction of the upper spleen due to the cutting of the short gastric arteries. In its treatment, it may be sufficient to provide analgesia. In our study, partial splenic infarction was observed only in one patient (0.06%). 

Rhabdomyolisis is a rare complication of major surgeries like bariatric surgeries. After injury of the scletal muscle, intracellular enzymes and myoglobin released. This causes hiperkalemia, hipoclasemia, disseminated intravascular coagulation and as a result of this clinic acute renal failure can be seen, and can complicated the bariatric surgery in the postoperative period. Mortality rate of the major surgeries increases with the levels 20% as a result of renal failure^21^. Prolonged surgery and severe obesity are important risc factors related with rhabdomyolisis. The incidence of rhabdomyolisis and acute renal injury after bariatric surgery is %1,4-22,7 and %1-3, respectively^14^. Nor Hanipah et al reported postoperative early remnal failure incidence as 0.9%^21^. For treatment of rhabdomyolisis, hidration is the main treatment option. But heamodialisis can be applicable in need^20^. The rhabdomyolisis rate and renal failure as a result of this pathology was seen in three patients (0,18 %) in our study. As a result of this aggressive fluid replacement (30-40 ml/kg) therapy, our renal failure rate was lower than the literature. Prefered fluid replacement options was 5% dextrose and isotonic sodium chloride. 

Since DeMaria first described the OS-MRS classification to determine the risk of postoperative mortality for bariatric surgery patients in 2007, hypothesis has been put forward that this classification can also be used in terms of postoperative complications^11^. Sarela et al. stated that bariatric surgery and OS-MRS classification are independent risk factors for postoperative complications^26^. In another study by Lorente et al., they evaluated 198 patients who underwent gastric band and sleeve gastrectomy, and they stated that OS-MRS classification is useful in the detection of patients at risk for complications in the postoperative period after bariatric surgery^17^. Orlowski et al stated that OS-MRS is beneficial in evaluating the development of postoperative complications and choosing appropriate bariatric surgery. However, the most important limitation in their studies was stated to be the low number of cases^23^. However, later on, in a study conducted by Garcia et al, they evaluated the correlation of OS-MRS with Clavien-Dindo surgical complication classification and stated that OS-MRS classification was not sufficient to predict morbidity and mortality preoperatively^15^. Similar to Garcia et al., we found that the perioperative complication risk before discharge did not show a statistically significant difference between the groups according to the OS-MRS classification. In addition, when it was evaluated in terms of major and minor complications according to Clavien-Dindo surgical complication classification, no statistically significant difference was observed between the OS-MRS classification groups. 

A similar study such as the evaluation of OS-MRS in terms of complication development described by DeMaria stated that the BMI value of 50 and above in patients with obesity surgery increased the risk of developing complications, in a study by Buchwald and Oien which determined that BMI is a predictor of postoperative complication^3^. Similar to these studies, in 2015, Aminian et al. reported in their studies that the BMI value is a risk factor for the development of perioperative complications to calculate risk in sleeve gastrectomy^1^. Sanni et al. also stated that each one-point increase in BMI value caused a 2% increase in the risk of complications in their studies evaluating perioperative complications^25^. Major et al also reported that having BMI values of 50 and above is a risk factor for postoperative complication development^18^. However, with an increase in clinical experience and the number of cases in high-volume clinics, it was revealed that the value of preoperative BMI does not relate to postoperative complications^6^. Similarly, in our study, there was no difference between the groups when the group with a BMI value of 40 and above was grouped according to each five-point increase. 

The most important limitation of our study is its retrospective character. In addition, due to the inability to evaluate the perioperative complications of the patients after discharge, the type of difference between the two early periods could not be examined. Therefore, in terms of treatment options and risk determination associated with weight loss, no evaluation could be made between these two perioperative periods. However, the strongest points of our study were the high patient volumes and the high discharge rates without complications.

Management of complications of LSG can be more successful with prospective randomized studies, in which two periods of early complications, before and after discharge, are compared.

## CONCLUSION

Sleeve gastrectomy is a type of bariatric surgery that can be successfully applied with low early complication rates before discharge. Although the number of perioperative complications before discharge is low, patients should be carefully evaluated and treatment options should be reviewed. OS-MRS classification is inadequate in determining early complications and major and minor complications before discharge. 
